# Thermal Stability Improvement of Core Material via High Internal Phase Emulsion Gels

**DOI:** 10.3390/polym15214272

**Published:** 2023-10-30

**Authors:** Jinhua Hu, Yongxue Liang, Xueyao Huang, Guangxue Chen, Dingrong Liu, Zhuangzhuang Chen, Zheng Fang, Xuelong Chen

**Affiliations:** 1 State Key Laboratory of Food Science and Resources, Jiangnan University, Wuxi 214122, China; wzadls@163.com (Y.L.); vivian200811@163.com (X.H.); 6200112005@stu.jiangnan.edu.cn (G.C.); 17368649529@163.com (D.L.); 6220112129@stu.jiangnan.edu.cn (Z.C.); 2 School of Food Science and Technology, Jiangnan University, Wuxi 214122, China; 3 State Key Laboratory of New Textile Materials and Advanced Processing Technology, School of Materials Science and Engineering, Wuhan Textile University, Wuhan 430200, China; 4 Atera Water Pte Ltd., 1 Corporation Drive, Singapore 619775, Singapore; xchen014@e.ntu.edu.sg

**Keywords:** thermal sensitive core materials, thermal stability, dynamic high-pressure microfluidization (HPM), high internal phase emulsion gel

## Abstract

Biocompatible particle-stabilized emulsions have gained significant attention in the biomedical industry. In this study, we employed dynamic high-pressure microfluidization (HPM) to prepare a biocompatible particle emulsion, which effectively enhances the thermal stability of core materials without the addition of any chemical additives. The results demonstrate that the HPM-treated particle-stabilized emulsion forms an interface membrane with high expansion and viscoelastic properties, thus preventing core material agglomeration at elevated temperatures. Furthermore, the particle concentration used for constructing the emulsion gel network significantly impacts the overall strength and stability of the material while possessing the ability to inhibit oxidation of the thermosensitive core material. This investigation explores the influence of particle concentration on the stability of particle-stabilized emulsion gels, thereby providing valuable insights for the design, improvement, and practical applications of innovative clean label emulsions, particularly in the embedding and delivery of thermosensitive core materials.

## 1. Introduction

High internal phase emulsion (HIPE), characterized by a dispersed phase volume fraction higher than 74%, has gained significant popularity across industries such as chemicals, food, pharmaceuticals, and petrochemicals. This innovative emulsion system offers great potential for the creation of advanced materials [1]. These materials possess diverse properties and find applications in various high-tech fields, impacting everyday lives through their use in filtration membranes, organic semiconductors, and tissue engineering scaffolds. Moreover, HIPE serves as an efficient vehicle for delivering active substances and facilitating sustained release in the domains of food and pharmaceuticals [2]. This characteristic presents numerous opportunities for development and innovation in these fields. When the continuous and/or dispersed phase of HIPE forms a gel, it is classified as a high internal phase emulsion gel (HIPE-gel).

There has been a growing interest in the application of particle emulsifiers for stabilizing emulsions, particularly those composed of hard inorganic particles like titanium dioxide [3] and silicon dioxide [4]. However, these particles often suffer from limited biodegradability and biocompatibility, which restricts their use in the food and pharmaceutical industries. Currently, particle emulsifiers have advanced from rigid particles to soft deformable particles possessing characteristics of both particles and polymeric particles [5]. The softness of these particles has a significant impact on the formation and stability of emulsions. Unlike hard particles, soft particles can spontaneously adsorb onto fluid interfaces, requiring less energy input [6]. Biocompatible soft particles primarily fall into two major biopolymer categories, i.e., proteins and polysaccharides, including commercialized food proteins such as whey protein [7,8], soy protein [9], and gelatin [10], as well as polysaccharides like starch [11] and cellulose [12]. For instance, successful stabilization of high internal phase emulsions (HIPE) was achieved to improve their resistance to aggregation using low-concentration gelatin particles (0.5%) [13]. Chitosan aggregates were utilized with varying degrees of cross-linking as particle emulsifiers to prepare HIPE with adjustable pore structures [14]. Wheat alcohol-soluble protein–chitosan conjugates were employed at low concentrations (0.5~2%) as particle emulsifiers to prepare HIPE with oil phase volume fractions as high as 83%, which exhibited favorable plasticity, viscoelasticity, curcumin protection, and resistance against oil oxidation [15].

Currently, the preparation of certain highly internal phase emulsions (HIPEs) still relies on the use of crosslinkers or organic solvents that do not meet clean label standards. These substances may persist in the emulsion products, severely limiting their applicability in the fields of food, pharmaceuticals, and health products. Hence, it is imperative to discover a more energy-efficient, environmentally friendly, and uncomplicated method to produce particle emulsifiers that are suitable for clean label applications, which presents a significant challenge. These particle emulsifiers possess exceptional interfacial properties, ensuring high safety and suitability for large-scale industrial production and commercialization [16]. One promising approach in HIPE research involves utilizing high-pressure homogenization, a technique capable of imparting proteins with novel structural and functional attributes through top-down (i.e., particle size reduction) or bottom-up (i.e., particle re-aggregation) processes [17]. This treatment enhances the interfacial properties of proteins, while also offering advantages such as ease of operation, scalability, reproducibility, and continuous processing. Consequently, high-pressure homogenization can be employed to physically modify particle emulsifiers for industrial production, making it an ideal technique for developing protein-based particle emulsifiers. For example, whey protein nano-gel particles created through high-pressure homogenization have successfully been used to formulate curcumin-loaded emulsion gels, exhibiting both a high loading efficiency and excellent resistance against light-induced degradation [18].

This study utilized milk protein concentrate (MPC) enriched with casein micelles as the emulsifier for the particles. It is worth noting that casein micelles possess a highly hydrated porous structure with a complex hierarchical arrangement [19,20,21,22]. To create particle emulsifiers that are suitable for clean label production and are characterized by exceptional interfacial functionality, we employed high-pressure homogenization (HPM) to modify the intricate hierarchical structure of natural particles. This modification resulted in a modified MPC that can be used as a clean-label particle emulsifier. Consequently, O/W HIPE (high internal phase emulsion) gels were prepared using only MPC as the emulsifying stabilizer and conjugated linoleic acid as a representative thermosensitive core material. Our primary objective was to examine the influence of MPC concentration on the preparation and stability of HIPE gels, as well as their capacity to hinder lipid oxidation during storage and improve the thermal stability of CLA.

## 2. Materials and Methods

### 2.1. Materials

MPC485 (protein 80.67%, lactose 5.72%, fat 1.63%) was purchased from Fonterra Co., Ltd. (Auckland, New Zealand). Conjugated linoleic acid (CLA) was purchased from Toole Biotechnology Co., Ltd. (Shenzhen, Guangdong, China). All the other chemicals of analytical grade were from Sinopharm Chemical Reagent Co., Ltd. (Shanghai, China). All the solutions were prepared using ultrapure water (Milli-Q Ultrapure Water Purification Systems, Billerica, MA, USA).

### 2.2. Preparation of MPC Dispersions under High Pressure

MPC is composed of whey proteins and caseins in a ratio of 1:4. Whey protein is mainly composed of β-lactoglobulin (β-Lg) and α-lactalbumin (α-La). Casein is mainly composed of four types of monomers, κ-casein, αs_2_-casein, αs_1_-casein, and β-casein, with a ratio 1.3:1:4:4 [23]. These monomers form micelles, where they are linked to each other by hydrophobic interactions and hydrogen bonds, and by colloidal calcium phosphates. Over 95% of the casein in milk is present in the casein micelles [24]. In addition, a small fraction of casein exists as monomers or small aggregates [25]. The MPC powder was dispersed in ultrapure water and stirred at room temperature for 2 h at 600 rpm, followed by an additional 10 h of stirring at 15 °C and 600 rpm. It is worth noting that the ultrapure water used in our experiments lacked trace elements and minerals, which may have led to slight differences in the size of casein micelles compared to natural micelles in milk. However, the MPC utilized in our research was an industrial product obtained through milk ultrafiltration and spray drying. Even when we attempted to reconstruct MPC using milk ultrafiltrate and simulated milk ultrafiltrate, we still could not achieve casein micelles identical to those found in natural milk. Furthermore, our aim was to make our study more aligned with practical production. Therefore, we chose ultrapure water as our research medium.

The MPC dispersion (5% weight percentage) was preheated to 50 °C and then subjected to three cycles of HPM using a high-pressure homogenizer equipped with a Z-shaped module containing microchannels with a diameter of 75 μm (Nano Disperser, Antos Nano Technology Limited Co., Suzhou, China). Each HPM treatment was conducted at 90 MPa. The temperature of the MPC dispersion at the outlet of the homogenizer was approximately 60 °C. Three replicates were prepared for each MPC dispersion treated with HPM at 90 MPa, labeled as MPC-HPM90. The untreated MPC dispersion served as the control and was labeled as MPC-0.

### 2.3. Preparation of HIPE-Gels

HIPE-gels were prepared with the oil phase volume fraction of 75%, utilizing conjugated linoleic acid (CLA) as the core material (oil phase) and an aqueous dispersion containing 2 or 4 wt% MPC (MPC-0, MPC-HPM90) as the aqueous phase. To facilitate visualization of the CLA using both visual and microscope observation, Nile red dye (0.1% *w*/*v*) was added to the CLA. First, 10 mL of protein dispersion was accurately measured and transferred into a 120 mL plastic beaker. Subsequently, 30 mL of CLA was slowly introduced to the aqueous phase using a constant flow pump, while high-speed shearing dispersion at a speed of 10,000 rpm (~4785× *g*) was conducted for 1 min with 10 s pauses using the high-speed disperser (T18, IKA, Staufen, Germany). It is worth noting that MPC is a multi-component mixture, and the particles mentioned in the article refer to the particle emulsifier. The resulting high internal phase emulsion gels (HIPE-gels) were obtained. The experiment was performed at a controlled temperature of 25 °C, utilizing a constant temperature water bath. Three replicates of the HIPE-gels were prepared. For storage, the HIPE-gels were maintained under controlled conditions of 45 °C in constant temperature shaking incubators within a light-free environment. The stability and lipid oxidation of the HIPE-gels were analyzed during a 14-day high-temperature storage period. Additionally, the samples were stored for an extended period of 7 weeks at 45 °C, allowing for visual assessment of their appearance and determination of shelf life. Approximately 8 g of the samples were stored in 10 mL glass vials for visual observation. For measuring other indicators during the storage period, 30 mL wide-mouth plastic dishes with covers were used.

### 2.4. Confocal Laser Scanning Microscope (CLSM)

Nile red, at a concentration of 0.1 g/L, was utilized to label the oil phase. A concave microscope slide was employed for immobilizing an appropriate volume of HIPE-gel, which was subsequently evenly spread and sealed with a coverslip. Imaging of the sample was carried out using a TCS SP8 laser confocal microscope (Leica, Weztlar, Germany) equipped with a 10× objective magnification and an excitation wavelength of 552 nm.

### 2.5. Rheological Analysis

A rotational rheometer (MCR92, Anton Paar, Graz, Germany) with a parallel plate having a diameter of 25 mm and a measurement gap of 1 mm was employed for the rheological analyses. The angular frequency was set to 10 rad/s, and amplitude scanning was conducted within a shear strain range of 0.01% to 1000%. Additionally, creep experiments were performed on the HIPE-gels by applying a stress of 10 Pa to the sample for 3 min, followed by a relaxation period of 3 min. The entire duration of changes in shear strain was recorded.

### 2.6. Lipid Oxidation Stability 

The stability of lipid oxidation was investigated by measuring the peroxide value (POV) of CLA in HIPE-gels based on the method of Zhuang et al. [26]. The FeCl_2_ solution used was freshly prepared. Freshly prepared or stored emulsion gel (0.1 g) was placed in a 15 mL centrifuge tube. A mixture of 3 mL of chloroform–methanol solution (2:1, *v*/*v*) was then added, and the mixture was vortexed for 30 s. The solution was mixed thoroughly for 20 min and then centrifuged at 3000× *g* for 10 min at room temperature. Precisely measured 1 mL of organic phase was then added to a 10 mL brown flask, followed by the addition of 50 μL of FeCl2 solution (3.5 g/L). The flask was filled with a chloroform–methanol solution (2:1, *v*/*v*), and 50 μL of KSCN solution (300 g/L) was added after mixing. After 5 min at room temperature, the absorbance of the solution was measured at 500 nm. The POV was calculated using Equation (1):

(1) $$\;X=\frac{c-c_{0}}{m\times 55.84\times 2\times \frac{V_{2}}{V_{1}}}$$

where *X* is the content of peroxides in the sample (meq/kg), *c* is the mass of iron in the sample obtained from the standard curve (μg), $c_{0}\;$ is the mass of the zero-tube iron obtained from the standard curve (μg), $V_{1}\;$ is the sample dilution total volume (mL), $V_{2}\;$ is the sample volume for measurement (mL), *m* is the mass of sample (g), 55.84 is the atomic weight of iron, and 2 is conversion factor.

### 2.7. Centrifugal Stability

The influence of a high temperature and centrifugal force on the physical stability of the O/W HIPE-gels was assessed using a LUMiSizer^®^ stability analyzer [27]. The gel samples were freshly prepared under room temperature conditions before centrifugation. During the testing process, parallel near-infrared light (865 nm) traversed the sample pool, allowing for the measurement of transmissivity as a function of the sample’s position. The testing conditions were maintained at 45 °C and 3000 rpm for a duration of 150 min. Once the tests were finished, a test result image (transmission–position image) was generated automatically for each sample. The red lines in each image indicate the modifications in the transmittance rate of different positions in the testing tube during the testing process.

### 2.8. Statistical Analysis

The results were expressed as the mean ± standard deviation. The statistical analyses was performed using IBM SPSS Statistics 25 (SPSS Inc., Chicago, IL, USA). An analysis of variance (ANOVA) was conducted, followed by Duncan’s test at a 95% confidence level. Statistical significance was determined at a *p*-value of less than 0.05, indicating significant differences between the samples.

## 3. Results and Discussion

### 3.1. Visual Observation of Prepared HIPE-Gels

**Figure 1:**
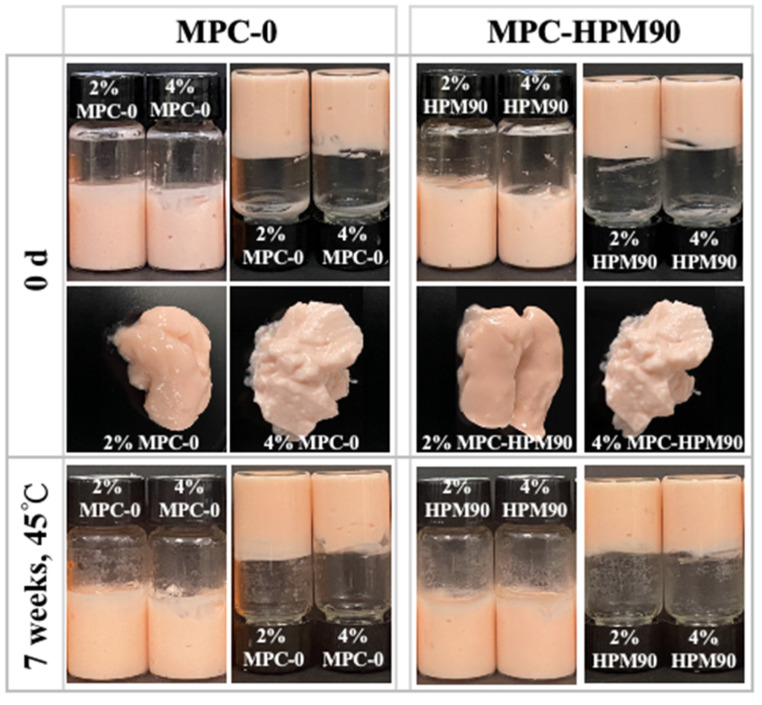
Visual observation of HIPE-gels during storage at 45 °C.

The prepared HIPEs were carefully placed in glass bottles and inverted for observation, as depicted in Figure 1.

The HIPEs were formulated using two different concentrations (2 wt%, 4 wt%) of MPC. These MPC concentrations tightly adhered to the glass bottles, exhibiting gel-like properties upon inversion, signifying the successful preparation of the HIPE-gels. In contrast, the HIPE-gels prepared using whey protein microgels with concentrations ranging from 2.5% to 7.5% exhibited low flowability, which was also observed in ref. [28], causing them to flow down to the bottom of the glass bottle during inversion. In our study, HIPE-gels prepared with a lower concentration (2 wt%) of MPC, regardless of whether the MPC underwent HPM treatment, displayed a softer texture and an observable oil sheen on the surface, suggesting slight oil phase leakage. Interestingly, the HIPE-gels formulated with a higher concentration (4 wt%) of MPC exhibited well-supported structures, indicating an improved strength of the network structure due to the elevated particle concentration. Furthermore, all the MPC-prepared HIPE-gels demonstrated excellent long-term stability, with no significant visual changes observed after seven weeks of storage at 45 °C (Figure S1, Supplementary Materials).

### 3.2. Microstructure of HIPE-Gels 

**Figure 2:**
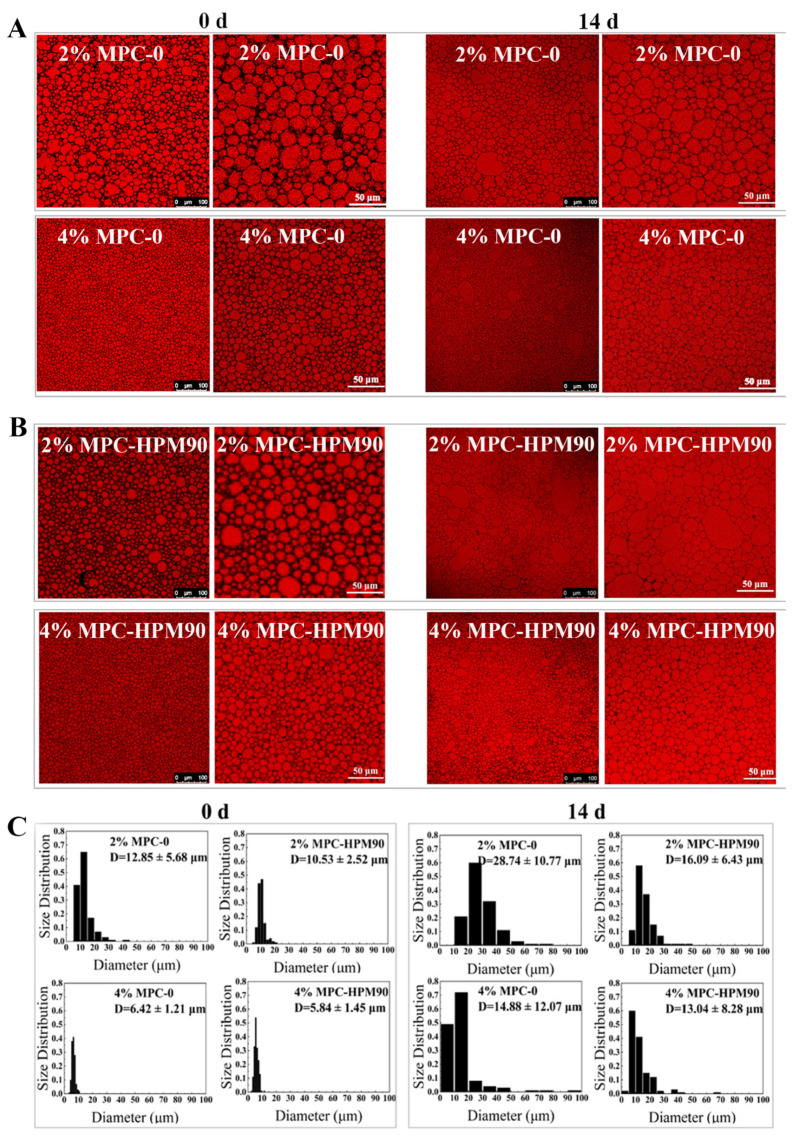
CLSM images (**A**,**B**) and the size distribution (**C**) of the oil drops in the HIPE-gels during storage at 45 °C.

To further investigate the influence of MPC particle concentration on the performance of the HIPE-gels, we employed confocal laser scanning microscopy (CLSM) to observe their microstructures. Furthermore, ImageJ software was utilized to analyze the droplet size and distribution based on the CLSM images obtained. As depicted in Figure 2, all the oil droplets in the HIPE-gels exhibited a densely packed arrangement, assuming polygonal shapes due to squeezing and deformation. This phenomenon can be ascribed to the dispersed phase volume fraction surpassing the requirement for close packing, which is consistent with prior research on HIPE-gels stabilized by whey protein isolate [28]. However, it is unavoidable that there may still be some circular shapes of varying sizes present.

The particle concentration of the MPC played a significant role in determining the size, morphology, and size distribution of the oil droplets in both the freshly prepared and stored HIPE-gels. In the HIPE-gels fabricated using a low concentration (2 wt%) of MPC, the oil droplets were larger, irregularly distributed, and displayed uneven shapes. Specifically, the average size of the oil droplets in the HIPE-gels prepared with MPC-0 was measured at 12.85 ± 5.68 μm, while those prepared with the MPC-HPM90 were slightly smaller, with an average size of 10.53 ± 2.52 μm. By increasing the MPC concentration to 4 wt%, the size of the oil droplets in the HIPE-gels decreased, and they assumed an irregular circular shape. The average size of the oil droplets in the HIPE-gels prepared using MPC-0 was 6.42 ± 1.21 μm, while those prepared using MPC-HPM90 exhibited an average size of 5.84 ± 1.45 μm. This relationship between particle concentration and droplet size can be attributed to the insufficiency of protein-based particle emulsifiers at lower concentrations, which fail to adequately cover the oil droplet surface during the shear dispersion process, resulting in droplet coalescence.

Additionally, the use of MPC with or without HPM treatment for HIPE-gel preparation influenced the morphology, size, and size distribution of the oil droplets. The incorporation of MPC-HPM90 as an emulsifier in the HIPE-gel formulation led to a noticeable improvement in the homogeneity of smaller oil droplets. This enhancement can be attributed to the increased deformability and collapsibility of the particles. The impact of this effect was most prominent in samples with lower concentrations (2 wt%), as evident from Figure 2. However, when the MPC concentration was increased to 4 wt%, the elevated quantity of protein particles allowed for complete coverage of the oil–water interface [28,29,30], thus overcoming differences in oil droplet morphology, size, and distribution caused by particle internal architecture. Consequently, any variations arising from the deformed particle shapes resulting from HPM treatment, which affected droplet characteristics, were successfully mitigated. Therefore, no significant differences were observed in the freshly prepared HIPE-gels with a higher MPC concentration, regardless of whether the HPM treatment was applied.

Following a two-week storage experiment at 45 °C, the concentration of the protein-based particle emulsifiers emerged as a crucial factor affecting the stability of the HIPE-gels. In the HIPE-gels stabilized using a low concentration (2 wt%) of MPC, severe droplet coalescence and enlargement were observed, resulting in large oil droplets reaching approximately 100 μm. Specifically, the average size of the oil droplets in the HIPE-gels prepared using MPC-0 was measured at 28.74 ± 10.77 μm, while those prepared using MPC-HPM90 exhibited a smaller average size of 16.09 ± 6.43 μm. Conversely, the droplet size experienced minimal changes during storage in the HIPE-gels stabilized with MPC-HPM90 at a high concentration (4 wt%), whereas some oil droplets aggregated in the HIPE-gels stabilized with MPC-0 (Figure 2). The average size of the oil droplets in the HIPE-gels prepared with MPC-0 was determined to be 14.88 ± 12.07 μm, whilst those prepared with MPC-HPM90 demonstrated an average size of 13.04 ± 8.28 μm. The complete coverage of the particles facilitated enhanced long-term stability of the HIPE-gels at elevated temperatures and displayed improved storage stability.

### 3.3. Dynamic Viscoelastic Properties of HIPE-Gels

**Figure 3:**
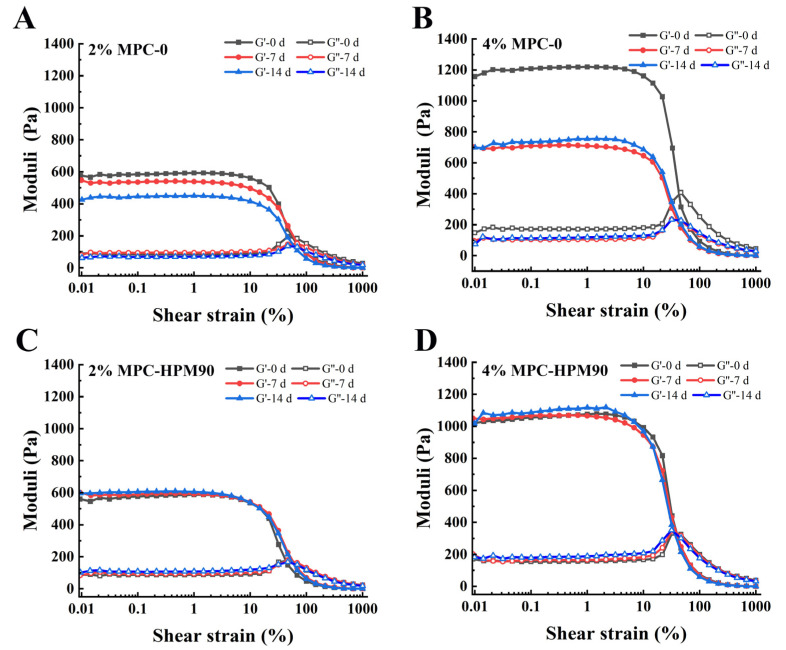
Amplitude sweep for the HIPE-gels during storage at 45 °C. Amplitude sweep for the HIPE-gels of 2% MPC-0 (**A**), 4% MPC-0 (**B**), 2% MPC-HPM90 (**C**) and 4% MPC-HPM90 (**D**).

The rheological properties of emulsions have a significant impact on their stability and practical applications. In order to gain a comprehensive understanding of the rheological properties of HIPE-gels and evaluate their practical utility, we conducted experiments on HIPE-gels prepared with different concentrations of MPC to examine their stability under large amplitude oscillatory shear. Figure 3 presents an investigation of the strain-dependent behavior of both the storage modulus (G′) and loss modulus (G′′) of the HIPE-gels, conducted at a fixed angular frequency of 10 rad/s.

Figure 3 shows the amplitude scanning of the HIPE-gels during storage at 45 °C. As can be observed from the figure, the concentration of MPC also affected the rheological behavior of the HIPE-gels. In the linear viscoelastic region, the G′ of the emulsion gels increased with an increase in the MPC concentration [14,31]. This suggests that as the concentration of the particle emulsifier increases, a more compact gel network structure could be formed inside the HIPE-gels, thereby increasing the mechanical strength of the HIPE-gels. In other words, increasing the MPC concentration could improve the mechanical strength and stability of the HIPE-gels. It has been suggested that an entangled protein network between adsorbed and non-adsorbed protein molecules is the major reason for the high elastic modulus and gel-like structure of emulsion gels [32]. Nevertheless, increasing the concentration of MPC-0 could not effectively improve the structural stability of the HIPE-gels during storage. Specifically, after 14 d of storage, the G′ of the HIPE-gels fabricated with 4 wt% MPC-0 decreased by approximately 41.67%. However, the HIPE-gels prepared with MPC-HPM90 exhibited excellent structural stability during storage. The improved performance of the HIPE-gels could be explained by the contribution of the HPM treatment, which enhances their ability to deform and collapse on the oil droplet surface [5]. The processing of HPM improves the deformation and collapse capacity of the MPC present on the oil droplet surface. Additionally, it strengthens the interpenetration between the particle emulsifiers, thereby leading to stronger protein interactions and the creation of a denser viscoelastic interface. This interface exhibits heightened mechanical capabilities coupled with prolonged HIPE-gel stability.

**Figure 4:**
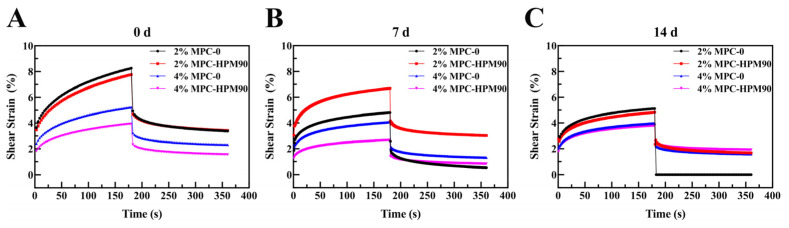
Creep–recovery behavior of the HIPE-gels during storage at 45 °C. Creep recovery behavior of the HIPE-gels during storage for 0 day (**A**), 7 days (**B**) and 14 days (**C**) at 45 °C.

The dynamic viscoelastic properties of the HIPE-gels were assessed using creep recovery tests, as depicted in Figure 4. Notably, all the tested samples underwent instantaneous deformation upon stress loading and unloading.

The strain value represents the cumulative deformation experienced by the gel sample under stress. As this value increased, the stiffness of the gel samples noticeably decreased, while a decrease in strain corresponded to an increase in gel hardness. Hence, the strain value serves as an indicator of gel firmness. When subjected to constant stress, the HIPE-gels stabilized using MPC-0 displayed higher strains compared to those stabilized using MPC-HPM90. This discrepancy suggests that the gels stabilized using MPC-0 were more susceptible to deformation due to their fragile gel structure, rendering them vulnerable to external perturbations. Conversely, the HIPE-gels stabilized using MPC-HPM90 exhibited minimal deformation when exposed to external force. This remarkable resistance to deformation can be attributed to the formation of interfacial membranes by deformed particles subsequent to the HPM treatment. MPC-HPM90 may optimize the benefits of both soft and hard particles when placed at the interface. Softer particles (e.g., casein micelles) can spontaneously adsorb on the interface, while harder particles (e.g., whey proteins and small aggregates of casein monomers) have limited adaptability [6,33]. Soft particles can adsorb spontaneously on the fluidic interface, while hard particles can constrain the lateral compression of soft particles during the divergent reaction. These viscoelastic membranes act as buffers, reducing the interaction between oil droplets and resulting in the formation of a stronger gel network. Consequently, such gels exhibit enhanced resistance to deformation. Figure 4 displays a general inverse relationship between the increase in shear strain and the extension of storage time, particularly after 7 days of storage. In both the aging and recovery steps, the shear strain obtained from the fresh-prepared HIPE-gels (0 day) was greater than that obtained from the aged HIPE-gels (7 days), suggesting that the aged HIPE-gels were unable to effectively respond to shear strain, resulting in increased stiffness. Regardless of whether the MPC underwent HPM treatment or not, the HIPE-gels prepared using 2 wt% MPC demonstrated a higher strain under the external force. This increased susceptibility to stress deformation in low particle concentration HIPE-gels can be attributed to their soft and flowable gel texture, as well as their flexible molecular network structure, as previously reported [34]. With an increase in particle concentration, the contact area between the particle emulsifier and the oil phase in the HIPE-gels also increased. Additionally, a higher concentration of the particle emulsifier led to more stable and stronger gel networks, thereby enhancing resistance to deformation. Consequently, the strain of the HIPE-gels gradually decreased at 4 wt% MPC, indicating that higher emulsifier concentrations contribute to stronger network structures within the gels.

### 3.4. Lipid Oxidation of HIPE-Gels under Elevated Temperatures

**Figure 5:**
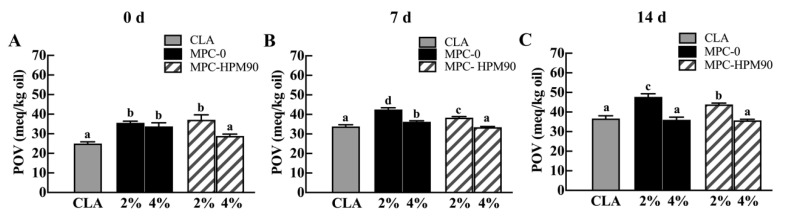
The lipid oxidation of CLA in HIPE-gels during storage at 45 °C. Different letters (a–d) indicate significant difference (*p* < 0.05) due to HPM treatment and different MPC concentration. The lipid oxidation of CLA in HIPE-gels during storage for 0 day (**A**), 7 days (**B**) and 14 days (**C**) at 45 °C.

Conjugated linoleic acid (CLA) is a type of polyunsaturated fatty acid [35] that undergoes rapid oxidation at high temperatures [36], making it an excellent model for studying thermal sensitivity in core materials. In our study, we investigated the effectiveness of HIPE (high internal phase emulsion) gels in delaying the lipid oxidation of CLA by increasing the temperature during this process. Figure 5 illustrates the impact of stable HIPE gels with varying particle concentrations as emulsifiers on the delayed oxidation of conjugated linoleic acid.

The peroxide value (POV) of pure and unprotected CLA was determined to be 25.00 ± 0.88 meq/kg oil. However, the POV of CLA increased when incorporated into the freshly prepared HIPE-gels. Notably, the oxidation degree of CLA was more pronounced in the HIPE-gels stabilized with 2 wt% MPC. The POV of CLA in the HIPE-gels stabilized using MPC-0 and MPC-HPM90 was measured at 35.60 ± 0.84 meq/kg oil and 37.14 ± 2.51 meq/kg oil, respectively. In the HIPE-gels stabilized with freshly prepared 2% MPC, regardless of whether MPC-0 or MPC-HPM90 was used, insufficient protein concentration resulted in incomplete coverage of the oil droplet surfaces. Consequently, lipid oxidation occurred during the approximately 20-min HIPE-gel preparation process. The level of lipid oxidation was similar in both samples, as depicted in Figure 5.

After 14 days of storage, the POV of pure CLA significantly increased to 36.69 ± 1.41 meq/kg oil. Meanwhile, the HIPE-gels stabilized using 2 wt% MPC exhibited an acceleration in peroxide production, as evident from the POV of CLA. In fact, the POV of CLA in the HIPE-gels stabilized using MPC-0 and MPC-HPM90 reached 47.71 ± 1.62 meq/kg oil and 43.88 ± 0.66 meq/kg oil, respectively. As the particle emulsifier concentration increased, the HIPE-gels effectively suppressed the lipid oxidation of CLA. Figure 5 demonstrates that the inhibition of lipid oxidation in the HIPE-gels stabilized with both 4% MPC-0 and 4% MPC-HPM90 was comparable, which was attributable to the formation of a continuous protein network on the surface of the oil droplets after 14 days of storage at 45 °C. This network hinders oxidants from coming into contact with the oil phase. In contrast, traditional emulsions with elevated water activity levels (>0.4) are expected to exhibit accelerated rates of lipid oxidation due to their O–W interface, which enlarges the surface area of oil droplets and facilitates interactions with the pro-oxidants present in the surrounding water phase [37]. Therefore, the oxidation rate of CLA in the HIPE-gels stabilized with 2 wt% MPC was found to be significantly higher than that observed in pure CLA oil.

Our findings reveal an intriguing phenomenon of inhibited CLA oxidation in response to an increased particle emulsifier concentration. This observation can be attributed to the three-dimensional network formed during gelation in HIPE-gels, which restricts the diffusion and transfer of oxygen, pro-oxidants, and free radicals to the O–W interface [38,39]. Consequently, lipid oxidation of CLA in HIPEs is delayed.

### 3.5. Physical Stability of HIPE-Gels under Elevated Temperatures

**Figure 6:**
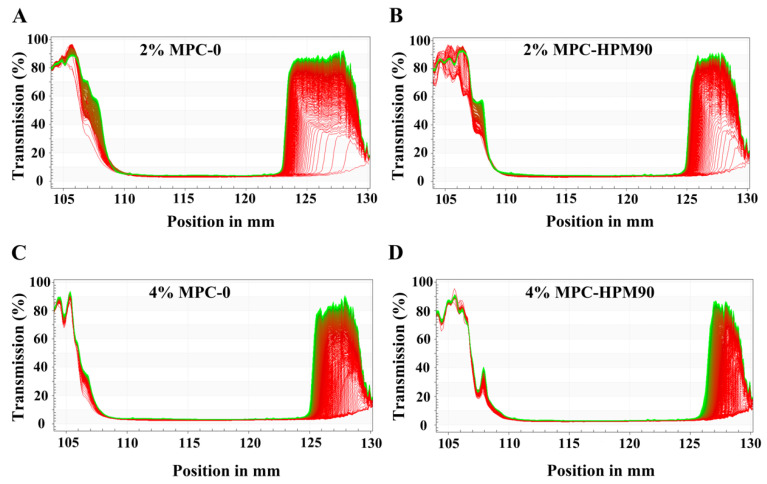
LUMISizer^®^ profiles of the HIPE-gels at 45 °C under 3000 rpm. The lines in each image indicate the modifications in the transmittance rate of different positions in the testing tube during the testing process. LUMISizer^®^ profiles of the HIPE-gels of 2% MPC-0 (**A**), 2% MPC-HPM90 (**B**), 4% MPC-0 (**C**) and 4% MPC-HPM90 (**D**) at 45 °C under 3000 rpm.

To assess the physical stability of the HIPE-gels, we utilized an accelerated centrifugation technique using LUMisizer® stability analysis (Figure 6). This method allows for evaluating stability under simulated mechanical forces and provides insights into long-term storage stability [40], as the centrifugal force accelerates flocculation and the coalescence of oil droplets [41].

In Figure 6, we present the integrated transmission curves of different HIPE-gels under 865 nm parallel near-infrared light over time. The position at 130 mm corresponds to the bottom of the HIPE-gel samples, while approximately 105 mm corresponds to the top. As the transmittance of emulsions is inversely proportional to the concentration of oil droplets, higher transmittance values indicate lower droplet concentrations, and vice versa. When using near-infrared light to detect the internal structures of gels, the initial transmittance is low due to the intact nature of the gel network. However, the application of centrifugal force breaks the gel network, leading to increased light transmittance. Oil droplets spill upwards, while water released from the disrupted gel structure settles down.

The majority of the initial profile lines (highlighted in red, Figure 6) in each figure were located at the base, indicating that the HIPE-gels originally possessed consistent, opaque gel structures with extremely low transmittance. After subjecting them to accelerated centrifugation at 3000 rpm for 150 min, the bottom part of the final profile lines (green lines) exhibited an increase in transmittance of 80–90%. This increase suggests that the HIPE-gel structure was disrupted under centrifugal force, leading to water seepage at the bottom and enhanced transmittance.

We observed that the HIPE-gels stabilized with lower concentrations (2 wt%) of MPC demonstrated a significant increase in transmittance near the bottom of the sample tube (Figure 6A,B), indicating their lower stability compared to those stabilized with higher concentrations (4 wt%) of MPC. As the concentration of MPC increased, the difference between the profiles recorded at different times during centrifugation decreased, suggesting an improved stability of the HIPE-gels. Several studies have evaluated emulsion stability using the LUMISizer^®^. For instance, Niu et al. [42] assessed the stability of O/W emulsions prepared using sugar beet pectin (SBP) (0.5–2.0%) and gum Arabic (GA) (0.5–15.0%) at various concentrations. Their findings demonstrated that both the SBP- and GA-stabilized emulsions became increasingly stable as the concentration rose. Su et al. [43] monitored transmission changes using a LUMISizer^®^ to evaluate the stability of HIPEs prepared using β-lactoglobulin-propylene glycol alginate composite hydrogel particles (β-lgPPs) at different concentrations (c = 0.1–2.0 wt%). The results indicated that HIPEs with high particle concentrations (c = 1.0–2.0 wt%) exhibited a relatively high physical stability, with an extremely slow creaming rate during centrifugation and a final value not exceeding 0.025.

Moreover, the HIPE-gels stabilized with MPC-HPM90 displayed less variation in transmission intensity at the same concentration. Among all the samples, the HIPE-gel stabilized using 4 wt% MPC-HPM90 exhibited minimal changes under accelerated centrifugation, indicating its high stability against coalescence, even in the presence of centrifugal force. Notably, the MPC-stabilized HIPE-gels demonstrated considerable resistance to droplet coalescence. Despite water seepage from the gel structure, no Nile red-stained oil droplets were released from the emulsion. Theoretical principles suggest that applying centrifugal force to a mixture of oil and water would result in phase separation, with the oil phase rising to the top. However, the HIPE-gels we prepared exhibited exceptional physical stability owing to the O–W interfacial membrane’s high mechanical strength, which was constructed by soft particles.

## 4. Conclusions

This study investigated the impact of the particle concentration of MPC modified with HPM on their own structure and thermal stability during storage, as well as its effect on the thermal stability of the encapsulated core materials. The results indicate that at lower concentrations (2%), the MPC particles failed to provide sufficient coverage to the oil droplets, resulting in a fragile network structure and increased possibility of oil droplet coalescence. In contrast, higher concentrations (4 wt%) of MPC demonstrated a notable ability to maintain long-term stable oil droplets. Specifically, the HIPE-gels stabilized using MPC-HPM90 exhibited insignificant changes in oil droplet size, size distribution, and gel network strength during a 14-day high-temperature storage period. These gels displayed excellent physical stability in elevated temperature environments and effectively inhibited lipid oxidation of the core material. This study provides valuable experimental data and theoretical guidance for the development, refinement, and application of particle emulsifiers. The research findings pave the way for the creation of more efficient emulsifiers that are suitable for use in environmentally friendly labeled consumer products. In summary, we have successfully developed HIPE-gels, a novel material that can enhance the thermal stability of heat-sensitive CLA without requiring any additional chemical emulsifiers. In future studies, we will investigate the feasibility of using HIPE-gels as a template for the preparation of porous materials and to polymerize lenoic acid. This technology has the potential to offer significant advantages over traditional emulsion polymerization processes, which use toxic surfactants that can have harmful environmental and health implications.

## Data Availability

The data presented in this study are available from the corresponding author upon request.

## Supplementary Materials

The following supporting information can be downloaded at: https://www.mdpi.com/article/10.3390/polym15214272/s1, Figure S1: The instability index of HIPE-gels.
