# Supplementary material for: Thermal Stability Improvement of Core Material via High Internal Phase Emulsion Gels
Source: Polymers (Basel). 2023 Oct 30;15(21):4272. doi: 10.3390/polym15214272 (PMC10647363; doi:10.3390/polym15214272)
Supplement: Supplementary file 1 [file polymers-15-04272-s001.zip › polymers-2663591-supplementary.pdf]

polymers

Supplementary Materials: Thermal stability improvement of core material via high internal phase emulsion gels

Jinhua Hu, Yongxue Liang, Xueyao Huang, Guangxue Chen, Dingrong Liu, Zhuangzhuang Chen, Zheng Fang and Xuelong Chen

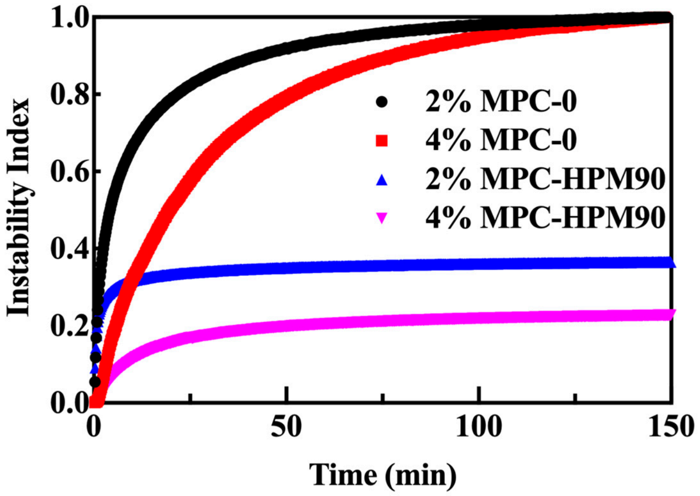

Figure S1. The instability index of HIPE-gels.
